# The Effect of α-Monolaurin and Butyrate Supplementation on Broiler Performance and Gut Health in the Absence and Presence of the Antibiotic Growth Promoter Zinc Bacitracin

**DOI:** 10.3390/antibiotics10060651

**Published:** 2021-05-29

**Authors:** Bakang R. Letlole, Ellen P. C. W. Damen, Christine Jansen van Rensburg

**Affiliations:** 1 Department of Animal Science, University of Pretoria, Pretoria 0002, South Africa; rletlole@sovfoods.co.za; 2 Research & Development, FRA^®^melco B.V., 4941 SB Raamsdonksveer, The Netherlands; E.Damen@framelco.com

**Keywords:** broilers, α-monolaurin, sodium butyrate, intestinal health, zinc bacitracin

## Abstract

The use of antibiotic growth promoters (AGP) is common practice to improve broiler production and performance. The use of AGP is under discussion as it can induce bacterial resistance. The purpose of this study was to determine the impact of removing AGP from broiler feed and study the effect of feed additives. For those countries where in-feed AGP are still permitted, the effect of the products in the presence of AGP was evaluated. Half the number of male broilers received a diet free of AGP, whereas the other half received a diet supplemented with zinc bacitracin at 0.5 g/kg. Both diets were either without additional additives or combined with a coated sodium butyrate, α-monolaurin or a combination of these additives. Raised under optimal conditions, the incorporation of AGP had no effect on broiler performance, but negatively affected villi height and villi height to crypt depth (VH:CD) ratio in the duodenum. In the absence of AGP, butyric acid and α-monolaurin had a positive effect on villi height. In the presence of AGP, α-monolaurin resulted in the lowest feed conversion ratio and improved VH:CD ratio in the duodenum, jejunum and ileum. Both feed additives had minimal effect on performance parameters but showed small positive effects on gut health in the absence of AGP and could play a role in the strategy to replace AGP.

## 1. Introduction

The Food and Agriculture Organization paints a bleak picture, reporting a deteriorated food security and raised numbers of malnutrition in many countries across the world [1]. To achieve sustainable production of nutritious and accessible food for the growing human population, a healthy livestock is of utmost importance. The use of in-feed antibiotics is common practice to improve broiler production and performance in several parts of the world. Multiple modes of action for the growth-promoting effect of antibiotics in livestock have been determined, including a reduction in nutrient losses to microorganisms colonizing the gastrointestinal tract (GIT), altering microbiome diversity, reducing growth inhibiting microbial metabolites and prevention of sub-clinical disease [2,3]. There is criticism of the use of in-feed antibiotics as its use in food-producing animals can cause pathogenic microorganisms to become resistant, making human and animal diseases hard to treat [4,5], presenting a serious threat to public health.

Intensive animal production systems often require viable alternative strategies for antibiotic growth promoters (AGP). Considering the modes of action of AGP, the pursuit for alternatives focuses on the GIT environment. Potential AGP alternatives must be able to reduce the number of pathogens, shift the microbiome towards more beneficial bacteria, [6] improve the efficiency of nutrient utilization by the growing animal and improve its immune response [7]. Derivatives of fatty acids such as sodium butyrate and α-monolaurin may be a solution.

Butyric acid, a short-chain aliphatic carboxylic fatty acid, is found naturally in plants and dairy products, and is produced in the GIT of birds by microbial fermentation of fiber or starch [8]. In broilers, sodium butyrate has been observed to improve intestinal development and growth rate, prevent colonization of different pathogens and modulate the immune response [9]. Recent research showed that in-feed butyrate supplements can indeed increase butyrate concentrations in the hindgut of broilers, which induced beneficial changes in the microbiome and provided protection against *Salmonella enteritidis* colonization [10]. Alpha-monolaurin is a monoester formed from glycerol and the medium-chain fatty acid lauric acid [11]. This molecule has antiviral, antibacterial, antifungal, anti-inflammatory and immune-modulating properties [11,12,13,14,15]. Recently, under Brazilian conditions it was shown that α-monolaurin improved broiler performance compared to in-feed zinc bacitracin [16].

Little research is available on α-monolaurin and coated butyric acid as potential AGP replacers under South African conditions. Therefore, the purpose of this study was to determine the impact of removing zinc bacitracin from broiler feed and to study the effect of two alternative products, a coated sodium butyrate and α-monolaurin, on broiler performance and gut health. For those countries such as South Africa where in-feed AGP are still permitted, the effect of these alternative products in the presence of zinc bacitracin was evaluated as well.

## 2. Results

### 2.1. Broiler Performance

The total mortality over the experimental period was 2.24%. No significant differences between any of the treatments were observed.

Weekly body weight (BW) is presented in Table 1. At day 14, the inclusion of AGP increased BW (*p* < 0.05). None of the feed additives had an effect on BW when AGP was present in the diet. However, in the absence of AGP, the inclusion of butyrate increased BW at day 7 and 14 (*p* < 0.05). Butyrate supplementation increased mean BW at day 7 compared to α-monolaurin (*p* < 0.05). At day 28, mean BW was higher with α-monolaurin supplemented to the diet compared to no additives or the combination of butyrate and α-monolaurin (*p* < 0.05).

Weekly and overall feed intake (FI) is shown in Table 2. None of the feed additives affected FI in AGP-rich diets. Compared to the negative control and the combination of feed additives, butyric acid alone increased FI between day 8–14, day 29–35 and day 0–35 in AGP-free diets (*p* < 0.05). Butyric acid also increased mean FI at day 29–35 and day 0–35 compared to α-monolaurin (*p* < 0.05), although α-monolaurin itself did not decrease feed intake.

Weekly and overall feed conversion ratio (FCR) is presented in Table 3. The use of AGP in the diets did not improve FCR. In diets containing AGP, the inclusion of α-monolaurin improved FCR at day 8–14, day 29–35 and from day 0–35 compared to the combination of α-monolaurin and butyric acid (*p* < 0.05). Mean FCR was improved from 0–35 when α-monolaurin was supplemented in the diet compared to butyric acid alone or in its combination (*p* < 0.05).

### 2.2. Gastro-Intestinal Development

Table 4 presents the effects of the studied feed additives on villi height in the absence and presence of AGP at 20 days of age. The inclusion of AGP in the diet reduced villi height in the jejunum (*p* < 0.05). At this age, duodenal villi height was improved by butyric acid, α-monolaurin and the combination of both products regardless of the inclusion of AGP (*p* < 0.05). The addition of α-monolaurin, alone and in combination with butyrate, improved villi height in the jejunum while butyric acid alone improved villi height in the ileum (*p* < 0.05) in AGP-rich diets. Table 5 presents the effect of the tested products on villi height at day 33 and shows that the presence of AGP reduced villi height in the duodenum and ileum (*p* < 0.05). Duodenal villi height was improved by α-monolaurin and the combination of both products regardless of the inclusion of AGP (*p* < 0.05), and by butyric acid in AGP-rich diets (*p* < 0.05). Alpha-monolaurin, alone and in combination with butyric acid, had a positive effect on jejunum villi height in AGP-free diets. Villi height in the ileum was improved by the combination of products in AGP-rich diets.

Table 6 presents the effects of the feed additives on crypt depth at day 20. The addition of AGP decreased crypt depth in the jejunum and ileum (*p* < 0.05). In the absence of AGP, the combination of products reduced crypt depth in the duodenum, jejunum and ileum. In the presence of AGP, crypt depth in the jejunum was increased by butyric acid, α-monolaurin and the combination of products at day 20. At day 33, the combination of feed additives decreased crypt depth in the jejunum and ileum in AGP-free diets (Table 7).

Table 8 presents the villi height to crypt depth (VH:CD) ratio of broilers at 20 days of age. The inclusion of AGP in the diet decreased mean VH:CD ratio in the duodenum and jejunum (*p* < 0.05). In the absence of AGP, butyric acid, α-monolaurin and the combination of both feed additives improved VH:CD ratio in the duodenum, jejunum and ileum (*p* < 0.05). In the presence of AGP, the combination of products improved VH:CD ratio in the jejunum (*p* < 0.05). Mean VH:CD ratio was improved by butyric acid, α-monolaurin and the combination in the duodenum and ileum, whereas only the combination of products improved mean VH:CD ratio in the jejunum at day 20 (*p* < 0.05). Table 9 presents the VH:CD ratio of broiler of 33 days of age. Supplementation of zinc bacitracin in the diet decreased the VH:CD ratio in the duodenum (*p* < 0.05). In AGP-free diets, the combination of additives improved VH:CD ratio in the duodenum and jejunum (*p* < 0.05). In AGP-rich diets, α-monolaurin improved VH:CD ratio in the duodenum, jejunum and ileum (*p* < 0.05). The combination of products increased VH:CD ratio in the duodenum and jejunum in AGP-rich diets (*p* < 0.05).

### 2.3. Goblet Cells

Table 10 presents the effects of feed additives in the absence and presence of AGP on the number of goblet cells at day 33. The inclusion of AGP in the diets reduced the mean number of goblet cells in the ileum (*p* < 0.05) and specifically in the presence of butyrate (*p* < 0.05). In the presence of AGP, no effect of the feed additives was observed. In the absence of AGP, the combination of products reduced the number of goblet cells in the duodenum (*p* < 0.05). Butyric acid increased the number of goblet cells in the absence of AGP in the jejunum and ileum (*p* < 0.05). When diets were supplemented with α-monolaurin or combined with butyric acid, the presence of AGP had an inhibitory effect on the number of goblet cells per 100 µm of villi in the duodenum (*p* < 0.05). This same effect was found in the ileum when the diet was supplemented with butyric acid (*p* < 0.05).

## 3. Discussion

In the present study, no effect of zinc bacitracin on broiler performance was found. Body weight, feed intake and FCR were not affected by the antibiotic growth promoter. This is in contrast with previous findings that showed clear growth-promoting effects and improved feed efficiency during the production cycle of broilers supplemented with zinc bacitracin [17,18,19]. The lack of improved growth performance in the present study may be due to the optimal rearing conditions. Broilers were raised in a controlled environment at low stocking density and with low pathogenic pressure, reaching a BW at day 35 well above male Ross 308 broiler performance objectives [20]. Furthermore, all diets contained a coccidiostat, which may have led to the lack of improvement when AGP was added to the diet compared to the negative control group. Despite this limitation of the trial, interesting observations were made on the effect of AGP on gut health and development. Shortening of the villi and a decreased VH:CD ratio were observed in the duodenum and jejunum when zinc bacitracin was added to the diet. Although it did not result in impaired animal performance, it indicates a possible harmful effect of zinc bacitracin on gut development under optimal conditions. Another study reported a positive effect of zinc bacitracin on broiler performance, whereas no effect was seen on villus height, crypt depth and VH:CD ratio in the duodenum, jejunum and ileum [21]. Al-Baadani et al. [22] added antibiotics (oxytetracycline and neomycin) to *Clostridium perfringens*-challenged broilers and found that villi of the ileum and jejunum were significantly shorter than in unmedicated birds. More research is needed to establish if there is a negative effect of zinc bacitracin on gut development.

In the absence of AGP, butyric acid increased BW at day 7 and day 14 compared to the negative control group. The effect of sodium butyrate on body weight is not unambiguously. El-Ghany et al. [23] reported an increase in weight gain of birds supplemented with sodium butyrate in both grower and finisher phases. This is in line with Sikander et al. [21] which found an increase in body weight during the 4th and 5th week of life. In contrast, Leeson et al. [24] and Jerzsele et al. [25] reported no significant difference in BW when butyric acid was supplemented in the starter, grower and finisher periods. Contrary to the present study, several studies [24,26,27,28] reported an improvement in FCR regardless of the inclusion level of butyric acid. The reduction in FCR was attributed to the improved digestion and absorption of nutrients as a result of improved gut development, increased pancreatic enzyme secretion and effects on gut mucosa, together with their antimicrobial activity [24,27,28,29]. Indeed, in the current study the presence of butyric acid increased duodenal villi height in both AGP-free and AGP-rich diets at day 20 and 33. Furthermore, butyric acid improved VH:CD ratio in the absence of zinc bacitracin in the duodenum, jejunum and ileum at day 20. This suggests an improved surface area for nutrient absorption, although this did not result in increased animal performance as animals were already performing at their genetic potential [17]. An increase in villi length by a coated sodium butyrate was expected. Enterocytes use butyric acid as an energy source, resulting in villi development. Butyric acid induces the gene expression and protein production of tight junctions, making the intestinal epithelial barrier less permeable for pathogens and toxins, which can contribute to intestinal health development and maturity [28]. In the jejunum and ileum, the administration of butyrate increased the mean number of goblet cells, specifically in AGP-free diets. Goblet cells are responsible for the mucus layer by secreting polymeric mucin glycoproteins. The mucus-gel layer is considered as the first line of defense that prevents foreign bacteria and pathogens from invading the intestinal mucosa [30,31]. The observed increase in goblet cells was expected since butyric acid has been reported to increase the production of mucins and host antimicrobial peptides [31,32]. Forder et al. [30] suggested that the presence of the neutral mucin in the jejunum and ileum was the result of increased intestinal maturity. Butyrate is known to have a positive effect on intestinal development, health and integrity which might explain the increase in the number of goblet cells when broilers are fed a form of butyric acid.

In the present study, no effect of α-monolaurin was found on BW in the absence or presence of zinc bacitracin. This is in contrast with the study of Fortuoso et al. [16], which showed that BW and average daily weight gain was higher in broilers receiving α-monolaurin compared to broilers receiving zinc bacitracin. That study is in line with the results of Mustafa [14] which showed an increased BW when the product FRA^®^ C12 Dry was fed at 2 or 4 kg/ton feed. In contrast, Liu et al. [33] did not find any effect of α-monolaurin on the growth of 49-day-old broilers. In the present study, no effect of α-monolaurin was found on feed intake. This in contrast with Fortuoso et al. [16], who found an increased feed intake in broilers receiving α-monolaurin to replace zinc bacitracin under Brazilian conditions. Mustafa [14] found a higher feed intake during the first and third week of age in broilers receiving FRA^®^ C12 Dry at 2 kg/ton feed. In these studies, the observed body weight of the control group was far below the performance objectives of the genetics [17,33], which indicates underlying problems that could be counteracted by α-monolaurin and result in improved broiler performance. In the present study, a positive effect of α-monolaurin was found on FCR. Fortuoso et al. [16] showed an improvement in FCR in a dose–response manner when α-monolaurin was fed. They suggested that the improvement in broiler performance could be related to the anti-pathogenic and anti-inflammatory effects of the molecule. Indeed, α-monolaurin can directly affect bacteria and viruses by disrupting the membrane or lipid envelope in their micellar state, resulting in bacterial death and inhibition of bacterial population growth, as well as causing inhibition of viral transmission and new viral infections [34,35]. Furthermore, the reduction in macrophage activity and pro-inflammatory cytokine production relieves the inflammation of the GIT [13,16] The reduction in pathogenic bacteria and the modulation of the immune response by α-monolaurin could save nutrients and energy which can be used for growth and intestinal development, reducing FCR. In the present study, α-monolaurin improved duodenal villi height at day 20 and 33 in the presence and absence of zinc bacitracin. At day 20, α-monolaurin improved villi height in the jejunum in the presence of AGP and at day 33 in the absence of AGP. Our observations are in line with previous published results that revealed that α-monolaurin tended to increase the VH:CD ratio of the duodenum and increased villi height and VH:CD ratio in the jejunum in yellow-feathered broilers [36].

On average, AGP decreased the numbers of goblet cells, which was found to be significant in the ileum. The number of goblets cells can give an indication about the maturity of the intestinal barrier. In a contrary perspective, bacterial infections can induce the intestinal mucin production for better protection [37]. Santin et al. [38] suggested that a reduction in the number of goblet cells may indicate that the gut is not exposed to stressing conditions. For example, lipopolysaccharides, the outer surface membrane component of Gram-negative bacteria, have been shown to increase the pro-inflammatory cytokine interleukin-8 (IL-8) and mucin (MUC) genes MUC5AC and MUC5b [39].

Antibiotic growth promoters and α-monolaurin both have the ability to reduce stressing conditions in the GIT by their anti-microbial and anti-inflammatory properties, and therefore may reduce mucin production. Our results show that the combination of α-monolaurin and butyric acid reduced numbers of goblet cells compared to butyric acid alone in AGP-free diets. Different cytokines have been reported to increase and change the mucin production and increase goblet cell proliferation [40]. In pigs, increased expression of pro-inflammatory cytokines IL-1β and IL-6 after *Escherichia coli* infection have been found [41], which was demonstrated to trigger mucin release and MUC gene expression [42,43]. Alpha-monolaurin has anti-inflammatory properties and has been shown to decrease the levels of pro-inflammatory cytokines such as IL-8, IL-1β and IL-6 during pathogenic infections in different in vitro models and in vivo trials [13,15,44]. The bactericidal and anti-inflammatory properties of α-monolaurin could have played a role in lowering the number of goblet cells when α-monolaurin and butyric acid were supplemented to an AGP-free diet compared to a diet which only contained butyrate. The group of birds supplemented with α-monolaurin with AGP had lower numbers of goblet cells in the duodenum compared to the α-monolaurin group of birds without AGP. Both α-monolaurin and AGP are anti-pathogenic and could have worked synergistically to reduce pro-inflammatory cytokines and therefore goblet cell formation in the duodenum. However, the mean numbers of goblet cells were numerically increased with α-monolaurin in the diet compared to no additives in the diets at each part of the GIT. This is in line with the results of Cui et al. [45], who found a significantly higher number of goblet cells in the jejunum of healthy piglets treated with α-monolaurin. They also suggest that the increased number of goblet cells indicates a more matured intestinal epithelium. More research is needed to determine the relationship between the number of goblet cells, GIT development and pathogenic infections.

This data shows that under very good conditions the use of zinc bacitracin had no effect on broiler performance as a growth promoter and had a negative effect on gut development. In the absence of AGP, butyric acid had a positive effect on BW in early life and positively affected gut development and maturity. In the absence of AGP, α-monolaurin mainly affected gut development. In the presence of AGP, butyric acid had no effect on broiler performance and on villi development which can be explained by the good rearing conditions, and possibly the presence of a coccidiostat in the diets, during this study. In the presence of AGP, α-monolaurin was shown to have the lowest FCR which is in line with an improved VH:CD ratio in the duodenum, jejunum and ileum. Both feed additives were shown to have positive effects on gut health, although a real synergistic relation could not be established in this study.

## 4. Materials and Methods

The study was approved by the Ethics Committee of the Faculty of Natural and Agricultural Sciences, University of Pretoria (EC170419-108). All animal procedures were conducted in an ethical manner and no birds were subjected to undue stress.

### 4.1. Broiler Husbandry

A total of 2304 one-day-old Ross 308 male broilers (Aviagen South Africa, Irene, South Africa) with an average body weight of 42.29 g were randomly distributed among 96 identical pens (24 birds per pen; 21.8 birds/m^2^) in a solid-sided broiler house near Uitenhage, South Africa. The facility was equipped with an electronic controller to manage temperature and ventilation by adjusting exhaust fan speed, coal boiler heaters and mist sprayers. The solid concrete floor of the house was covered with fresh wood shavings to the depth of 40 mm. Each pen contained one tube feeder and four nipple drinkers of which the height was continually adjusted to accommodate broiler growth. Birds had ad libitum access to feed and water throughout the experimental period. The initial house temperature of 35 °C prior to placement was gradually lowered to approximately 27 °C at 10 days of age and was kept constant until 35 days of age. The lighting program was set at 23L:1D from 1 to 7 days, 18L:6D from 8 to 21 days, 20L:4D from 22 to 31 days and finally 22L:2D on 32 and 33 days and 24L:0D on the last day before slaughter. The trial lasted for 35 days.

### 4.2. Feed and Experimental Design

Three maize–soybean basal diets were fed according to age. The starter diet was fed from 0 to 14 days of age in a crumble form and the grower and finisher diets were fed as pellets from 15 to 28 days and 29 to 35 days of age, respectively. Feed was formulated to meet the nutrient requirements of Ross 308 broilers [17] and reflected typical commercial diets in South Africa. Feed samples were analyzed for crude protein, moisture, ash, crude fiber, ether extract, Ca and total P, Na and K concentrations according to the methods described by the Association of Official Analytical Chemists [46]. Feed ingredient and nutrient concentrations of the basal diets are shown in Table 11 and Table 12.

A complete randomized block design with eight dietary treatments in a 2 × 4 arrangement was applied in the study. Two levels of an antibiotic growth promoter (zinc bacitracin 15%) were included, either at 0 or 0.5 g/kg, either with no additional feed additives, or combined with a coated sodium butyrate (Novyrate^®^ C), α-monolaurin (FRA^®^ C12 Dry) or both additives. Birds were randomly distributed over the 8 dietary treatments with 12 repetitions of each of the 24 animals. The treatment group only containing zinc bacitracin served as the positive control, while the negative control did not contain any of the tested feed additives nor zinc bacitracin. Novyrate^®^ C, containing 32% coated sodium butyrate and approximately 25% butyric acid, was obtained from Innovad (Essen, Belgium). Novyrate^®^ C was dosed at 1 g/kg in the starter feed, 0.75 g/kg in the grower feed and 0.25 g/kg in the finisher feed. FRA^®^ C12 Dry was obtained from FRAmelco B.V. (Raamsdonksveer, The Netherlands). FRA^®^ C12 Dry is a mixture of mono-, di- and triglycerides of lauric acid on a silica carrier with α-monolaurin as the main ingredient. FRA^®^ C12 Dry was dosed at 1 g/kg during the entire trial.

### 4.3. Measurements and Tissue Sampling

Body weight and FI per pen were recorded weekly and mortality as it occurred. Average bird BW, FI and FCR were calculated accordingly. Dead birds were weighed to adjust FCR for mortality. On day 20 and 33, two birds per pen with a BW close to the average pen BW were euthanized by cervical dislocation. For intestinal morphological examination, sections of the intestine (duodenum, jejunum and ileum) were cut into 2 cm pieces and stored in 10% neutral buffered formalin. Cross-sections measuring approximately 1 cm were prepared, enclosed in tissue cassettes and fixed in 10% neutral buffered formalin over 24 h. The staining method of Brancroft (2003) was adapted for this experiment. Each tissue sample was cut into a 4 to 5 µm section and placed onto a glass slide and stained with hematoxylin and eosin (H&E) and Alcian Blue/Periodic Acid Schiff (PAS). The H&E-stained intestinal sections were used to measure the villus height and crypt depth. Villus height was measured as the length between the villus–crypt axis and the tip of the villus. The crypt depth was measured from the villous–crypt axis to the base of the specific crypt. Villi height to crypt depth ratio was calculated accordingly. The PAS stain provided a clear, purple/blue image that was used to determine the number of neutral mucin goblet cells on the villi. The goblet cells were counted per 100 µm of villi. Slides were viewed and photographed using the Zeiss Axiovert 200 microscope (Carl Zeiss (Pty) Ltd., Johannesburg) with Axio Vision Rel. 4.8.2 software.

### 4.4. Statistical Analysis

The data on performance and gut morphology were analyzed as a complete randomized block design. Repeated measures of analysis of variation using the generalized linear model (GLM) procedure of the Statistical Analysis System (SAS, version 9.4) were performed on growth performance traits and included the effects of feed additive, with and without AGP. Each pen replication served as an experimental unit. Significance of differences between means was evaluated using the Fischer’s Least Significant Difference (LSD) test at a 95% confidence interval. Differences were accepted as significant when *p* < 0.05.

The linear model used is described by the following equation:

Y_ijk_ = µ + T_i_ + L_j_ + B_k_ + TL_ij_ + e_ijk_

where

Y = independent variable studied during the period;

µ = overall mean of the population;

T = effect of ith treatment;

L = effect of jth level;

TL = effect of the ijth interaction between treatment and level;

B = effect of kth block;

e = error associated with each dependent variable Y.

## Figures and Tables

**Table 1:** The effect of butyric acid and α-monolaurin, with and without the inclusion of an antibiotic growth promoter, on weekly body weight (g) of broilers (± standard error of the mean).

Period	Treatment	Without AGP ^α^	With AGP ^α^	Mean
Day 0	No additives	42.27 (±0.09)	42.34 (±0.09)	42.31 (±0.06)
	Butyric acid ^#^	42.39 (±0.09)	42.25 (±0.09)	42.32 (±0.06)
	Monolaurin *	42.28 (±0.09)	42.26 (±0.09)	42.27 (±0.06)
	Butyric acid ^#^ + Monolaurin *	42.26 (±0.09)	42.28 (±0.09)	42.27 (±0.06)
	Mean	42.30 (±0.04)	42.28 (±0.04)	
Day 7	No additives	186.43 ^b^ (±2.31)	191.52 (±2.31)	188.98 ^ab^ (±1.63)
	Butyric acid ^#^	193.17 ^a^ (±2.31)	193.69 (±2.31)	193.43 ^a^ (±1.63)
	Monolaurin *	187.96 ^ab^ (±2.31)	189.12 (±2.31)	188.54 ^b^ (±1.63)
	Butyric acid ^#^ + Monolaurin *	190.77 ^ab^ (±2.31)	192.16 (±2.31)	191.47 ^ab^ (±1.63)
	Mean	189.58 (±1.15)	191.62 (±1.15)	
Day 14	No additives	489.75 ^b1^ (±3.91)	501.15 ^2^ (±3.91)	495.45 ^b^ (±2.77)
	Butyric acid ^#^	503.79 ^a^ (±3.91)	506.45 (±3.91)	505.12 ^a^ (±2.77)
	Monolaurin *	496.69 ^ab^ (±3.91)	503.54 (±3.91)	500.11 ^ab^ (±2.77)
	Butyric acid ^#^ + Monolaurin *	498.39 ^ab^ (±3.91)	496.87 (±3.91)	497.63 ^ab^ (±2.77)
	Mean	497.16 (±1.96)	502.00 (±1.96)	
Day 21	No additives	1089.58 (±9.71)	1092.69 (±9.71)	1091.13 (±6.87)
	Butyric acid ^#^	1090.87 (±9.71)	1106.07 (±9.71)	1098.47 (±6.87)
	Monolaurin *	1090.74 (±9.71)	1100.15 (±9.71)	1095.45 (±6.87)
	Butyric acid ^#^ + Monolaurin *	1095.00 (±9.71)	1086.64 (±9.71)	1090.82 (±6.87)
	Mean	1091.55 (±4.86)	1096.39 (±4.86)	
Day 28	No additives	1731.80 (±13.55)	1750.60 (±13.55)	1741.20 ^b^ (±9.58)
	Butyric acid ^#^	1742.71 (±13.55)	1774.25 (±13.55)	1758.48 ^ab^ (±9.58)
	Monolaurin *	1764.95 (±13.55)	1776.23 (±13.55)	1770.59 ^a^ (±9.58)
	Butyric acid ^#^ + Monolaurin *	1736.45 (±13.55)	1748.33 (±13.55)	1742.39 ^b^ (±9.58)
	Mean	1743.98 (±6.77)	1762.35 (±6.77)	
Day 35	No additives	2436.13 (±22.54)	2467.31 (±22.54)	2451.72 (±15.94)
	Butyric acid ^#^	2465.89 (±22.54)	2491.28 (±22.54)	2478.58 (±15.94)
	Monolaurin *	2469.47 (±22.54)	2498.76 (±22.54)	2484.12 (±15.94)
	Butyric acid ^#^ + Monolaurin *	2440.57 (±22.54)	2447.79 (±22.54)	2444.18 (±15.94)
	Mean	2453.01 (±11.27)	2476.28 (±11.27)	

^α^ Antibiotic growth promoter: zinc bacitracin 15% (0.5 g/kg); ^#^ Novyrate^®^ C (starter 1 g/kg, grower 0.75 g/kg, finisher 0.25 g/kg); * FRA^®^ C12 Dry (1 g/kg); ^a,b^ column means per day with a distinctive superscript differ significantly (*p* < 0.05); ^1,2^ row means with a distinctive superscript differ significantly (*p* < 0.05).

**Table 2:** The effect of butyric acid and α-monolaurin, with and without the inclusion of an antibiotic growth promoter, on weekly and overall feed intake of broilers (± standard error of the mean).

Period	Treatment	Without AGP ^α^	With AGP ^α^	Mean
Day 0–7	No additives	152.73 (±1.96)	154.59 (±1.96)	153.66 (±1.38)
	Butyric acid ^#^	156.41 (±1.96)	157.87 (±1.96)	157.14 (±1.38)
	Monolaurin *	155.21 (±1.96)	153.72 (±1.96)	154.46 (±1.38)
	Butyric acid ^#^ + Monolaurin *	158.11 (±1.96)	155.86 (±1.96)	156.98 (±1.38)
	Mean	155.61 (±0.98)	155.51 (±0.98)	
Day 8–14	No additives	390.54 ^b^ (±3.75)	391.51 (±3.75)	391.02 ^b^ (±2.65)
	Butyric acid ^#^	402.14 ^a^ (±3.75)	397.64 (±3.75)	399.89 ^a^ (±2.65)
	Monolaurin *	397.66 ^ab^ (±3.75)	393.10 (±3.75)	395.38 ^ab^ (±2.65)
	Butyric acid ^#^ + Monolaurin *	388.34 ^b^ (±3.75)	394.10 (±3.75)	391.22 ^b^ (±2.65)
	Mean	394.67 (±1.87)	394.09 (±1.87)	
Day 15–21	No additives	858.25 (±15.24)	828.90 (±15.24)	843.57 (±10.78)
	Butyric acid ^#^	844.12 (±15.24)	832.51 (±15.24)	838.31 (±10.78)
	Monolaurin *	836.34 (±15.24)	843.22 (±15.24)	839.78 (±10.78)
	Butyric acid ^#^ + Monolaurin *	841.23 (±15.24)	812.37 (±15.24)	826.80 (±10.78)
	Mean	844.98 (±7.62)	829.25 (±7.62)	
Day 22–28	No additives	967.05 (±14.99)	978.38 (±14.99)	972.72 (±10.60)
	Butyric acid ^#^	983.43 (±14.99)	969.90 (±14.99)	976.67 (±10.60)
	Monolaurin *	966.10 (±14.99)	967.47 (±14.99)	966.79 (±10.60)
	Butyric acid ^#^ + Monolaurin *	946.50 ^1^ (±14.99)	992.65 ^2^ (±14.99)	969.58 (±10.60)
	Mean	965.77 (±7.50)	977.10 (±7.50)	
Day 29–35	No additives	1360.77 ^b^ (±25.76)	1409.19 (±25.76)	1384.98 ^ab^ (±18.21)
	Butyric acid ^#^	1443.41 ^a^ (±25.76)	1422.01 (±25.76)	1432.71 ^a^ (±18.21)
	Monolaurin *	1372.37 ^ab^ (±25.76)	1376.30 (±25.76)	1374.33 ^b^ (±18.21)
	Butyric acid ^#^ + Monolaurin *	1370.85 ^b1^ (±25.76)	1438.91 ^2^ (±25.76)	1404.88 ^ab^ (±18.21)
	Mean	1386.85 (±12.88)	1411.60 (±12.88)	
Day 0–35	No additives	3729.33 ^b^ (±34.36)	3762.56 (±34.36)	3745.95 ^ab^ (±24.30)
	Butyric acid ^#^	3829.50 ^a^ (±34.36)	3782.28 (±34.36)	3805.89 ^a^ (±24.30)
	Monolaurin *	3727.68 ^b^ (±34.36)	3733.81 (±34.36)	3730.74 ^b^ (±24.30)
	Butyric acid ^#^ + Monolaurin *	3705.03 ^b^ (±34.36)	3792.82 (±34.36)	3748.92 ^ab^ (±24.30)
	Mean	3747.89 (±17.18)	3767.87 (±17.18)	

^α^ Antibiotic growth promoter: zinc bacitracin 15% (0.5 g/kg); ^#^ Novyrate^®^ C (starter 1 g/kg, grower 0.75 g/kg, finisher 0.25 g/kg); * FRA^®^ C12 Dry (1 g/kg); ^a,b^ column means per period with a distinctive superscript differ significantly (*p* < 0.05); ^1,2^ row means with a distinctive superscript differ significantly (*p* < 0.05).

**Table 3:** The effect of butyric acid and α-monolaurin, with and without the inclusion of an antibiotic growth promoter (AGP), on weekly and overall feed conversion ratio of broilers (± standard error of the mean).

Period	Treatment	Without AGP ^α^	With AGP ^α^	Mean
Day 0–7	No additives	1.06 (±0.01)	1.04 (±0.01)	1.05 (±0.01)
	Butyric acid ^#^	1.04 (±0.01)	1.05 (±0.01)	1.04 (±0.01)
	Monolaurin *	1.07 (±0.01)	1.05 (±0.01)	1.06 (±0.01)
	Butyric acid ^#^ + Monolaurin *	1.06 (±0.01)	1.04 (±0.01)	1.05 (±0.01)
	Mean	1.06 (±0.01)	1.04 (±0.01)	
Day 8–14	No additives	1.29 (±0.01)	1.26 ^ab^ (±0.01)	1.28 (±0.01)
	Butyric acid ^#^	1.29 (±0.01)	1.27 ^ab^ (±0.01)	1.28 (±0.01)
	Monolaurin *	1.28 (±0.01)	1.25 ^a^ (±0.01)	1.27 (±0.01)
	Butyric acid ^#^ + Monolaurin *	1.26 (±0.01)	1.29 ^b^ (±0.01)	1.28 (±0.01)
	Mean	1.28 (±0.01)	1.27 (±0.01)	
Day 15–21	No additives	1.31 (±0.03)	1.36 (±0.03)	1.34 (±0.02)
	Butyric acid ^#^	1.33 (±0.03)	1.30 (±0.03)	1.31 (±0.02)
	Monolaurin *	1.32 (±0.03)	1.32 (±0.03)	1.32 (±0.02)
	Butyric acid ^#^ + Monolaurin *	1.30 (±0.03)	1.32 (±0.03)	1.31 (±0.02)
	Mean	1.31 (±0.02)	1.32 (±0.03)	
Day 22–28	No additives	1.49 (±0.03)	1.46 (±0.03)	1.48 ^ab^ (±0.02)
	Butyric acid ^#^	1.48 (±0.03)	1.45 (±0.03)	1.47 ^ab^ (±0.02)
	Monolaurin *	1.44 (±0.03)	1.42 (±0.03)	1.43 ^a^ (±0.02)
	Butyric acid ^#^ + Monolaurin *	1.51 (±0.03)	1.47 (±0.03)	1.49 ^b^ (±0.02)
	Mean	1.48 (±0.02)	1.45 (±0.02)	
Day 29–35	No additives	1.80 (±0.04)	1.84 ^ab^ (±0.04)	1.82 (±0.03)
	Butyric acid ^#^	1.87 (±0.04)	1.86 ^ab^ (±0.04)	1.87 (±0.03)
	Monolaurin *	1.88 (±0.04)	1.79 ^a^ (±0.04)	1.83 (±0.03)
	Butyric acid ^#^ + Monolaurin *	1.82 ^1^ (±0.04)	1.93 ^b2^ (±0.04)	1.88 (±0.03)
	Mean	1.84 (±0.02)	1.85 (±0.02)	
Day 0–35	No additives	1.48 (±0.01)	1.47 ^ab^ (±0.01)	1.48 ^ab^ (±0.01)
	Butyric acid ^#^	1.51 (±0.01)	1.47 ^a^ (±0.01)	1.49 ^b^ (±0.01)
	Monolaurin *	1.47 (±0.01)	1.44 ^a^ (±0.01)	1.46 ^a^ (±0.01)
	Butyric acid ^#^ + Monolaurin *	1.47 ^1^ (±0.01)	1.51 ^b2^ (±0.01)	1.49 ^b^ (±0.01)
	Mean	1.48 (±0.01)	1.48 (±0.01)	

^α^ Antibiotic growth promoter: zinc bacitracin 15% (0.5 g/kg); ^#^ Novyrate^®^C (starter 1 g/kg, grower 0.75 g/kg, finisher 0.25 g/kg); * FRA^®^ C12 Dry (1 g/kg); ^a,b^ column means per period with a distinctive superscript differ significantly (*p* < 0.05); ^1,2^ row means with a distinctive superscript differ significantly (*p* < 0.05).

**Table 4:** The effect of the butyric acid and α-monolaurin, with and without the inclusion of antibiotic growth promoters, on the villi height (µm) of the duodenum, jejunum and ileum of broilers at 20 days of age (± standard error of the mean).

Intestinal Segments	Treatment	Without AGP ^α^	With AGP ^α^	Mean
Duodenum	No additives	2829.66 ^b^ (±75.24)	2708.57 ^b^ (±75.24)	2769.12 ^b^ (±53.20)
	Butyric acid ^#^	3188.21 ^a^ (±75.24)	2976.97 ^a^ (±75.24)	3082.59 ^a^ (±53.20)
	Monolaurin *	3219.30 ^a1^ (±75.24)	2938.28 ^a2^ (±75.24)	3078.79 ^a^ (±53.20)
	Butyric acid ^#^ + Monolaurin *	3170.11 ^a1^ (±75.24)	2893.01 ^a2^ (±75.24)	3031.56 ^a^ (±53.20)
	Mean	3101.82 ^1^ (±37.62)	2879.21 ^2^ (±37.62)	
Jejunum	No additives	1770.21 ^1^ (±74.03)	1472.10 ^c2^ (±74.03)	1621.16 ^b^ (±52.35)
	Butyric acid ^#^	1808.52 ^1^ (±74.03)	1642.83 ^bc2^ (±74.03)	1752.67 ^bc^ (±52.35)
	Monolaurin *	1937.13 ^1^ (±74.03)	1687.65 ^ab2^ (±74.03)	1812.39 ^ac^ (±52.35)
	Butyric acid ^#^ + Monolaurin *	1964.48 (±74.03)	1888.85 ^a^ (±74.03)	1926.66 ^a^ (±52.35)
	Mean	1870.09 ^1^ (±37.01)	1672.85 ^2^ (±37.01)	
Ileum	No additives	1064.83 (±40.49)	983.93 ^b^ (±40.49)	1024.38 ^b^ (±28.63)
	Butyric acid ^#^	1164.02 (±40.49)	1146.42 ^a^ (±40.49)	1155.22 ^a^ (±28.63)
	Monolaurin *	1091.94 (±40.49)	1087.94 ^ab^ (±40.49)	1089.94 ^ab^ (±28.63)
	Butyric acid ^#^ + Monolaurin *	1172.10 (±40.49)	1090.91 ^ab^ (±40.49)	1131.50 ^a^ (±28.63)
	Mean	1123.22 (±20.25)	1077.30 (±20.25)	

^α^ Antibiotic growth promoter: zinc bacitracin 15% (0.5 g/kg); ^#^ Novyrate^®^C (starter 1 g/kg, grower 0.75 g/kg, finisher 0.25 g/kg); * FRA^®^ C12 Dry (1 g/kg); ^a–c^ column means per intestinal segment with a distinctive superscript differ significantly (*p* < 0.05); ^1,2^ row means with a distinctive superscript differ significantly (*p* < 0.05).

**Table 5:** The effect of butyric acid and α-monolaurin, with and without the inclusion of antibiotic growth promoters, on the villi height (µm) of the duodenum, jejunum and ileum of broilers at 33 days of age (± standard error of the mean).

Intestinal Segments	Treatment	Without AGP ^α^	With AGP ^α^	Mean
Duodenum	No additives	2989.78 ^b1^ (±86.23)	2595.58 ^b2^ (±86.23)	2792.68 ^b^ (±60.97)
	Butyric acid ^#^	3187.82 ^b1^ (±86.23)	2906.90 ^a2^ (±86.23)	3047.36 ^a^ (±60.97)
	Monolaurin *	3462.50 ^a1^ (±86.23)	3086.72 ^a2^ (±86.23)	3274.61 ^c^ (±60.97)
	Butyric acid ^#^ + Monolaurin *	3486.65 ^a1^ (±86.23)	3074.47 ^a2^ (±86.23)	3280.56 ^c^ (±60.97)
	Mean	3281.69 ^1^ (±43.11)	2915.92 ^2^ (±43.11)	
Jejunum	No additives	1591.21 ^b^ (±83.88)	1806.88 (±83.88)	1699.05 ^b^ (±59.32)
	Butyric acid ^#^	1822.23 ^ab^ (±83.88)	1799.30 (±83.88)	1810.76 ^ab^ (±59.32)
	Monolaurin *	1983.43 ^a1^ (±83.88)	1700.20 ^2^ (±83.88)	1841.81 ^ab^ (±59.32)
	Butyric acid ^#^ + Monolaurin *	1966.81 ^a^ (±83.88)	1812.18 (±83.88)	1889.50 ^a^ (±59.32)
	Mean	1840.92 (±41.94)	1779.64 (±41.94)	
Ileum	No additives	1375.34 ^1^ (±63.72)	1157.70 ^b2^ (±63.72)	1266.52 (±45.06)
	Butyric acid ^#^	1367.42 (±63.72)	1330.12 ^ab^ (±63.72)	1348.77 (±45.06)
	Monolaurin *	1320.83 (±63.72)	1260.44 ^ab^ (±63.72)	1290.63 (±45.06)
	Butyric acid ^#^ + Monolaurin *	1197.11 ^1^ (±63.72)	1380.94 ^a2^ (±63.72)	1289.03 (±45.06)
	Mean	1315.17 (±31.86)	1282.30 (±31.86)	

^α^ Antibiotic growth promoter: zinc bacitracin 15% (0.5 g/kg); ^#^ Novyrate^®^C (starter 1 g/kg, grower 0.75 g/kg, finisher 0.25 g/kg); * FRA^®^ C12 Dry (1 g/kg); ^a–c^ column means per intestinal segment with a distinctive superscript differ significantly (*p* < 0.05); ^1,2^ row means with a distinctive superscript differ significantly (*p* < 0.05).

**Table 6:** The effect of the butyric acid and α-monolaurin, with and without the inclusion of antibiotic growth promoters, on crypt depth (µm) of the duodenum, jejunum and ileum of broilers at 20 days of age (± standard error of the mean).

Intestinal Segments	Treatment	Without AGP ^α^	With AGP ^α^	Mean
Duodenum	No additives	407.78 ^a^ (±15.29)	382.36 (±15.29)	395.07 (±10.81)
	Butyric acid ^#^	389.59 ^ab^ (±15.29)	408.30 (±15.29)	398.94 (±10.81)
	Monolaurin *	370.00 ^ab1^ (±15.29)	419.44 ^2^ (±15.29)	394.72 (±10.81)
	Butyric acid ^#^ + Monolaurin *	364.23 ^b^ (±15.29)	402.25 (±15.29)	383.24 (±10.81)
	Mean	382.90 (±7.64)	403.09 (±7.64)	
Jejunum	No additives	361.38 ^a1^ (±13.22)	306.37 ^b2^ (±13.22)	333.88 (±9.35)
	Butyric acid ^#^	300.71 ^b1^ (±13.22)	355.50 ^a2^ (±13.22)	328.10 (±9.35)
	Monolaurin *	335.64 ^ab^ (±13.22)	362.92 ^a^ (±13.22)	349.28 (±9.35)
	Butyric acid ^#^ + Monolaurin *	310.76 ^b^ (±13.22)	344.23 ^a^ (±13.22)	327.50 (±9.35)
	Mean	327.12 (±6.61)	342.25 (±6.61)	
Ileum	No additives	387.64 ^a1^ (±14.98)	321.34 ^2^ (±14.98)	354.49 ^a^ (±10.59)
	Butyric acid ^#^	332.41 ^b^ (±14.98)	330.35 (±14.98)	331.38 ^ab^ (±10.59)
	Monolaurin *	272.25 ^c1^ (±14.98)	337.57 ^2^ (±14.98)	304.91 ^b^ (±10.59)
	Butyric acid ^#^ + Monolaurin *	306.87 ^bc^ (±14.98)	297.06 (±14.98)	301.96 ^b^ (±10.59)
	Mean	324.79 (±7.49)	321.58 (±7.49)	

^α^ Antibiotic growth promoter: zinc bacitracin 15% (0.5 g/kg); ^#^ Novyrate^®^C (starter 1 g/kg, grower 0.75 g/kg, finisher 0.25 g/kg); * FRA^®^ C12 Dry (1 g/kg); ^a,b^ column means per intestinal segment with a distinctive superscript differ significantly (*p* < 0.05); ^1,2^ row means with a distinctive superscript differ significantly (*p* < 0.05).

**Table 7:** The effect of the butyric acid and α-monolaurin, with and without the inclusion of antibiotic growth promoters (AGP), on crypt depth (µm) of the duodenum, jejunum and ileum of broilers at 33 days of age (± standard error of the mean).

Intestinal Segments	Treatment	Without AGP ^α^	With AGP ^α^	Mean
Duodenum	No additives	364.16 (±48.57)	425.80 (±48.57)	394.98 (±34.35)
	Butyric acid ^#^	356.92 (±48.57)	382.52 (±48.57)	369.72 (±34.35)
	Monolaurin *	352.21 (±48.57)	314.11 (±48.57)	333.16 (±34.35)
	Butyric acid ^#^ + Monolaurin *	288.33 ^1^ (±48.57)	434.25 ^2^ (±48.57)	361.29 (±34.35)
	Mean	340.40 (±24.29)	389.17 (±24.29)	
Jejunum	No additives	376.84 ^a^ (±33.15)	327.45 ^ab^ (±33.15)	352.14 (±23.44)
	Butyric acid ^#^	308.37 ^ab^ (±33.15)	296.56 ^ab^ (±33.15)	302.47 (±23.44)
	Monolaurin *	341.36 ^ab1^ (±33.15)	239.68 ^b2^ (±33.15)	290.52 (±23.44)
	Butyric acid ^#^ + Monolaurin *	278.79 ^b^ (±33.15)	351.37 ^a^ (±33.15)	315.08 (±23.44)
	Mean	326.34 (±16.57)	303.77 (±16.57)	
Ileum	No additives	316.49 ^a^ (±19.49)	275.44 (±19.49)	295.96 ^a^ (±13.79)
	Butyric acid ^#^	276.11 ^ab^ (±19.49)	278.11 (±19.49)	277.11 ^ab^ (±13.79)
	Monolaurin *	279.09 ^ab^ (±19.49)	232.29 (±19.49)	255.69 ^b^ (±13.79)
	Butyric acid ^#^ + Monolaurin *	229.70 ^b^ (±19.49)	271.04 (±19.49)	250.37 ^b^ (±13.79)
	Mean	275.35 (±9.75)	264.22 (±9.75)	

^α^ Antibiotic growth promoter: zinc bacitracin 15% (0.5 g/kg); ^#^ Novyrate^®^C (starter 1 g/kg, grower 0.75 g/kg, finisher 0.25 g/kg); * FRA^®^ C12 Dry (1 g/kg); ^a,b^ column means per intestinal segment with a distinctive superscript differ significantly (*p* < 0.05); ^1,2^ row means with a distinctive superscript differ significantly (*p* < 0.05).

**Table 8:** The effect of butyric acid and α-monolaurin, with and without the inclusion of antibiotic growth promoters, on VH:CD ratio of the duodenum, jejunum and ileum of broilers at 20 days of age (± standard error of the mean).

Intestinal Segments	Treatment	Without AGP ^α^	With AGP ^α^	Mean
Duodenum	No additives	6.98 ^b^ (±0.35)	7.21 (±0.35)	7.10 ^b^ (±0.25)
	Butyric acid ^#^	8.37 ^a^ (±0.35)	7.41(±0.35)	7.89 ^a^ (±0.25)
	Monolaurin *	8.72 ^a1^ (±0.35)	7.17 ^2^ (±0.35)	7.94 ^a^ (±0.25)
	Butyric acid ^#^ + Monolaurin *	8.77 ^a1^ (±0.35)	7.32 ^2^ (±0.35)	8.04 ^a^ (±0.25)
	Mean	8.21 ^1^ (±0.17)	7.28 ^2^ (±0.17)	
Jejunum	No additives	4.98 ^b^ (±0.30)	4.95 ^ab^ (±0.30)	4.96 ^b^ (±0.21)
	Butyric acid ^#^	6.17 ^a1^ (±0.30)	4.62 ^b2^ (±0.30)	5.39 ^ab^ (±0.21)
	Monolaurin *	5.89 ^a1^ (±0.30)	4.68 ^b2^ (±0.30)	5.29 ^b^ (±0.21)
	Butyric acid ^#^ + Monolaurin *	6.37 ^a^ (±0.30)	5.56 ^a^ (±0.30)	5.97 ^a^ (±0.21)
	Mean	5.85 ^1^ (±0.15)	4.95 ^2^ (±0.15)	
Ileum	No additives	2.80 ^b^ (±0.20)	3.21 (±0.20)	3.01 ^b^ (±0.14)
	Butyric acid ^#^	3.62 ^a^ (±0.20)	3.50 (±0.20)	3.56 ^a^ (±0.14)
	Monolaurin *	4.04 ^a1^ (±0.20)	3.27 ^2^ (±0.20)	3.66 ^a^ (±0.14)
	Butyric acid ^#^ + Monolaurin *	3.91 ^a^ (±0.20)	3.73 (±0.20)	3.82 ^a^ (±0.14)
	Mean	3.59 (±0.10)	3.43 (±0.10)	

^α^ Antibiotic growth promoter: zinc bacitracin 15% (0.5 g/kg); ^#^ Novyrate^®^C (starter 1 g/kg, grower 0.75 g/kg, finisher 0.25 g/kg); * FRA^®^ C12 Dry (1 g/kg); ^a,b^ column means per intestinal segment with a distinctive superscript differ significantly (*p* < 0.05); ^1,2^ row means with a distinctive superscript differ significantly (*p* < 0.05).

**Table 9:** The effect of butyric acid and α -monolaurin, with and without the inclusion of antibiotic growth promoters, on VH:CD ratio of the duodenum, jejunum and ileum of broilers at 33 days of age (± standard error of the mean).

Intestinal Segments	Treatment	Without AGP ^α^	With AGP ^α^	Mean
Duodenum	No additives	8.79 ^b1^ (±0.62)	6.47 ^b2^ (±0.62)	7.63 ^b^ (±0.44)
	Butyric acid ^#^	9.22 ^b^ (±0.62)	7.93 ^b^ (±0.62)	8.58 ^b^ (±0.44)
	Monolaurin *	9.97 ^b^ (±0.62)	10.21 ^a^ (±0.62)	10.09 ^a^ (±0.44)
	Butyric acid ^#^ + Monolaurin *	12.48 ^a1^ (±0.62)	9.15 ^a2^ (±0.62)	10.82 ^a^ (±0.44)
	Mean	10.12 ^1^ (±0.31)	8.44 ^2^ (±0.31)	
Jejunum	No additives	4.98 ^b^ (±0.43)	5.11 ^b^ (±0.43)	5.04 ^b^ (±0.30)
	Butyric acid ^#^	6.16 ^ab^ (±0.43)	6.29 ^ab^ (±0.43)	6.22 ^a^ (±0.30)
	Monolaurin *	5.87 ^b1^ (±0.43)	7.23 ^a2^ (±0.43)	6.55 ^a^ (±0.30)
	Butyric acid ^#^ + Monolaurin *	7.18 ^a^ (±0.43)	6.36 ^a^ (±0.43)	6.77 ^a^ (±0.30)
	Mean	6.05 (±0.21)	6.25 (±0.21)	
Ileum	No additives	4.65 (±0.38)	4.44 ^b^ (±0.38)	4.54 ^b^ (±0.27)
	Butyric acid ^#^	5.24 (±0.38)	5.20 ^ab^ (±0.38)	5.22 ^ab^ (±0.27)
	Monolaurin *	4.87 (±0.38)	5.78 ^a^ (±0.38)	5.33 ^a^ (±0.27)
	Butyric acid ^#^ + Monolaurin *	5.35 (±0.38)	5.10 ^ab^ (±0.38)	5.23 ^ab^ (±0.27)
	Mean	5.03 (±0.19)	5.13 (±0.19)	

^α^ Antibiotic growth promoter: zinc bacitracin 15% (0.5 g/kg); ^#^ Novyrate^®^C (starter 1 g/kg, grower 0.75 g/kg, finisher 0.25 g/kg); * FRA^®^ C12 Dry (1 g/kg); ^a,b^ column means per intestinal segment with a distinctive superscript differ significantly (*p* < 0.05); ^1,2^ row means with a distinctive superscript differ significantly (*p* < 0.05).

**Table 10:** The effect of butyric acid and α -monolaurin, with and without the inclusion of an antibiotic growth promoters, on the number of goblet cells per 100 µm of villi in the duodenum, jejunum and ileum of broilers at 33 days of age (± standard error of the mean).

Intestinal Segments	Treatment	Without AGP ^α^	With AGP ^α^	Mean
Duodenum	No additives	10.72 ^a^ (±0.534)	10.22 (±0.534)	10.47 (±0.377)
	Butyric acid ^#^	11.31 ^a^ (±0.534)	10.27 (±0.534)	10.79 (±0.377)
	Monolaurin *	12.05 ^a1^ (±0.534)	9.80 ^2^ (±0.534)	10.92 (±0.377)
	Butyric acid ^#^ + Monolaurin *	9.16 ^b1^ (±0.534)	10.77 ^2^ (±0.534)	9.96 (±0.377)
	Mean	10.81 (±0.267)	10.26 (±0.267)	
Jejunum	No additives	10.37 ^b^ (±0.633)	9.98 (±0.633)	10.13 ^b^ (±0.488)
	Butyric acid ^#^	12.76 ^a^ (±0.633)	11.03 (±0.633)	11.89 ^a^ (±0.488)
	Monolaurin *	11.58 ^ab^ (±0.633)	10.82 (±0.633)	11.20 ^ab^ (±0.488)
	Butyric acid ^#^ + Monolaurin *	10.59 ^b^ (±0.633)	10.74 (±0.633)	10.66 ^ab^ (±0.488)
	Mean	11.32 (±0.317)	10.62 (±0.317)	
Ileum	No additives	11.45 ^b^ (±0.533)	10.32 (±0.533)	10.89 ^b^ (±0.377)
	Butyric acid ^#^	12.97 ^a1^ (±0.533)	11.42 ^2^ (±0.533)	12.20 ^a^ (±0.377)
	Monolaurin *	11.71 ^ab^ (±0.533)	10.80 (±0.533)	11.25 ^ab^ (±0.377)
	Butyric acid ^#^ + Monolaurin *	10.86 ^b^ (±0.533)	11.41 (±0.533)	11.14 ^ab^ (±0.377)
	Mean	11.75 ^1^ (±0.267)	10.99 ^2^ (±0.267)	

^α^ Antibiotic growth promoter: zinc bacitracin 15% (0.5 g/kg); ^#^ Novyrate^®^C (starter 1 g/kg, grower 0.75 g/kg, finisher 0.25 g/kg); * FRA^®^ C12 Dry (1 g/kg); ^a,b^ column means per intestinal segment with a distinctive superscript differ significantly (*p* < 0.05); ^1,2^ row means with a distinctive superscript differ significantly (*p* < 0.05).

**Table 11:** Feed composition (%) of the basal diets.

Ingredients (%)	Starter	Grower	Finisher
Maize (yellow)	55.1	61.0	65.2
Soybean oilcake meal (46.5%)	34.6	29.7	26.1
Sunflower oilcake meal (36%)	4.00	4.00	4.00
Soya oil	2.20	2.10	1.90
Limestone	1.63	1.33	1.21
Mono-di-calcium phosphate	1.09	0.55	0.36
Salt (fine)	0.13	0.12	0.12
Lysine (HCl 78%)	0.28	0.30	0.30
Methionine (DL 98%)	0.29	0.27	0.24
Threonine (98%)	0.05	0.06	0.06
Sodium bicarbonate	0.28	0.31	0.32
Axtra Phy 100 g/t SK	0.01	0.01	0.01
Choline CL (60%)	0.20	0.20	0.20
Robenidine HCL (6.6%)	0.05	0.05	0.05
Broiler premix	0.15	0.15	0.10

**Table 12:** Calculated and analyzed composition (g/kg) of the basal diets.

Nutrient Composition (g/kg)	Starter Calculated	Starter Analyzed	Grower Calculated	Grower Analyzed	Grower Calculated	Grower Analyzed
Dry matter	883.4	887.7	882.8	883.7	882.4	886.4
AME (MJ/kg)	11.30		11.65		11.85	
Moisture	116.6	112.3	117.2	116.3	117.6	113.6
Crude protein	225	213	207	202	194	187
Fat	51.7	53.5	51.5	49.3	50.1	49.5
Crude fiber	35.5	49.3	35.4	39.8	35.3	40.5
Ash	60.9	51.5	50.4	42.6	45.6	41.8
Lysine ^1^	11.6		10.6		10.2	
Methionine	5.7		5.4		5.1	
TSAA ^2^	8.8		8.2		7.6	
Threonine	7.4		6.9		6.4	
Tryptophan	2.2		2.0		1.8	
Isoleucine	8.2		7.5		6.9	
Arginine	13.4		12.1		11.2	
Valine	9.0		8.2		7.7	
Glycine and serine	17.2		15.7		14.6	
Calcium	10.5	8.85	8.4	7.1	7.6	6.4
Total phosphorus	6.7	6.0	5.3	4.62	4.7	4.4
Sodium	1.6	1.24	1.6	1.28	1.6	1.26
Potassium	11	8.36	9.9	7.8	9.1	7.6

^1^ All amino acid values are given as digestible amino acid values for poultry; ^2^ TSAA: total sulphur-containing amino acids.

## Data Availability

Data sharing is not applicable to this article.

## Abbreviations

AGP	Antibiotic growth promoters
BW	Body weight
FCR	Feed conversion ratio
FI	Feed intake
GIT	Gastrointestinal tract
GLM	Generalized linear model
MUC	Mucin
SAS	Statistical Analysis System
